# Blimp-1 signaling pathways in T lymphocytes is essential to control the *Trypanosoma cruzi* infection-induced inflammation

**DOI:** 10.3389/fimmu.2023.1268196

**Published:** 2023-10-16

**Authors:** Luciana Benevides, Lais A. Sacramento, Franciele Pioto, Gabriel Dessotti Barretto, Vanessa Carregaro, João S. Silva

**Affiliations:** ^1^ Fiocruz-Bi-Institutional Translational Medicine Plataform, Ribeirão Preto, SP, Brazil; ^2^ Department of Biochemistry and Immunology Ribeirão Preto Medical School University of São Paulo, Ribeirão Preto, SP, Brazil

**Keywords:** BLIMP-1, T. cruzi, Th1 activating, cytotoxic CD8 + T cells, parasite replication

## Abstract

In many infectious diseases, the pathogen-induced inflammatory response could result in protective immunity that should be regulated to prevent tissue damage and death. In fact, in *Trypanosoma cruzi* infection, the innate immune and the inflammatory response should be perfectly controlled to avoid significant lesions and death. Here, we investigate the role of Blimp-1 expression in T cells in resistance to *T. cruzi* infection. Therefore, using mice with Blimp-1 deficiency in T cells (CKO) we determined its role in the controlling parasites growth and lesions during the acute phase of infection. Infection of mice with Blimp-1 ablation in T cells resulted failure the cytotoxic CD8^+^ T cells and in marked Th1-mediated inflammation, high IFN-γ and TNF production, and activation of inflammatory monocyte. Interestingly, despite high nitric-oxide synthase activation (NOS-2), parasitemia and mortality in CKO mice were increased compared with infected WT mice. Furthermore, infected-CKO mice exhibited hepatic lesions characteristic of steatosis, with significant AST and ALT activity. Mechanistically, Blimp-1 signaling in T cells induces cytotoxic CD8^+^ T cell activation and restricts parasite replication. In contrast, Blimp-1 represses the Th1 response, leading to a decreased monocyte activation, less NOS-2 activation, and, consequently preventing hepatic damage and dysfunction. These data demonstrate that *T. cruzi*-induced disease is multifactorial and that the increased IFN-γ, NO production, and dysfunction of CD8^+^ T cells contribute to host death. These findings have important implications for the design of potential vaccines against Chagas disease.

## Introduction

In most infectious diseases, the inflammatory response triggered by pathogens leads to the development of protective immunity. However, it is crucial to carefully regulate the immune response to prevent tissue damage and mortality (1). *Trypanosoma cruzi* infection, caused by an intracellular protozoan parasite, the etiological agent of Chagas disease, requires control of the infection through the innate and adaptive immune response (2–4). These responses can ensure host survival by limiting parasite growth and tissue lesions. Additionally, the adaptive immune response, involving both humoral and cell-mediated components, must be tightly regulated to prevent autoimmune responses (5, 6), which can lead to neuronal destruction and myocarditis, resulting in severe infectious illness affecting millions of people in Latin America (7). The control of parasite growth within nucleated cells relies on pro-inflammatory cytokines such as IFN-γ and TNF, as well as chemokines (8), which activate inducible nitric oxide synthase (iNOS) and promote nitric oxide production (9). However, while Th1 and Th17 cells are crucial for controlling the parasite, their excessive activation can exacerbate the inflammatory response, leading to tissue damage and organ dysfunction (10).

The B lymphocyte-induced maturation protein 1 (Blimp-1) is a transcription factor that plays a critical role in regulating the function of B and T lymphocytes (11–13). In various models of inflammatory diseases, such as asthma and colitis, mice with specific deletion of the Blimp-1 gene in T lymphocytes exhibit heightened cytokine production, which contributes to worsened inflammation (14, 15). However, the role of Blimp-1 in regulating T cell responses in Chagas disease remains unknown. In other parasitic diseases, such as malaria, visceral leishmaniasis (16), and African trypanosomiasis (17), Blimp-1 promotes the production of IL-10 by Tr1 cells, resulting in inflammation control. Therefore, our aim is to investigate the potential involvement of Blimp-1 within the T cell compartment in the pathogenesis of Chagas disease. By utilizing mice with T cell-specific deficiency of Blimp-1, we demonstrate the significance of this transcription factor in restricting parasite replication through cytotoxic CD8^+^ T cell activation, suppressing the Th1 response, and inhibiting the activation of inflammatory monocytes that produce TNF and nitric oxide. Additionally, Blimp-1 protects mice from the development of *T. cruzi* infection-induced metabolic disorders, hepatic damage, and dysfunction.

## Materials and methods

### Animals

Conditional Blimp-1^fl/fl^CD4^Cre^ (T cell-specific Blimp-1 deficient mice-CKO) and C57BL/6 Blimp-1^fl/fl^ (WT) generated by crossing C57BL/6 CD4^Cre^ mice were obtained from The Jackson Laboratory. All mice were genotyped, both for CRE and Flox, following the Jax mice instructions (stock number 017336 and 008100) and bred in the animal facility at the University of São Paulo, Brazil, maintained in a pathogen-free environment. Groups of 6 females of WT and CKO mice, age of 7-8 weeks, were used. All procedures were performed in the accordance with the International Guidelines for the Use of Animals and approved by the local Ethics Committee at the University of São Paulo, Brazil (123/2017).

### Mouse infection and treatments

For *in vivo* experiments, mice were inoculated intraperitoneally with 10^2^ or 10^3^ bloodstream trypomastigote forms of the Y strain. From day 5 after infection, blood parasitemia was evaluated in 5 μl of blood from the tail vein by counting 100 fields through direct observation under an optical microscope. Groups of mice infected with 100 trypomastigote forms were treated by gavage with 100 mg/Kg of Benznidazole (BNZ) or vehicle on days 7 and 9-post infection (pi) or intraperitoneally (ip) with dexamethasone (1 mg/Kg) or vehicle on days 9, 12 and 15 pi. The treatment with aminoguanidine were performed ip, daily, for 7 days, with 2.5 mg/Kg of aminoguanidine (AG) or vehicle. The survival rate and weight loss were assessed daily. The quantification of tissue parasitism was done as previously described (18).

### Tregs differentiation and adoptive transfer

Naive CD4^+^ (CD44^-^CD25^-^GFP^-^) T cells from Foxp3-GFP reporter mice were sorted from the spleen and lymph node cell suspensions using a FACSAriaII (BD Bioescience). CD4^+^ T cells were stimulated with plate-bound anti-CD3 (2ug/mL), anti-CD28 (1ug/mL) (both from BioXCell), IL-2 (100 U/mL), TGF-β (5 ng/mL), anti-IFN-γ, and anti-IL-4 (10 ug/mL) for 4 days in RPMI-1640 medium supplemented with 5% FBS (Gibco), 100 U/mL penicillin, 100 ug/mL streptomycin, 1 mM sodium pyruvate, nonessential amino acids, L-glutamine, and 50 uM 2-mercaptoethanol. All recombinant cytokines were obtained from RD, and neutralizing antibodies from BioXCell. After 4 days of culture, 2 x 10^6^ Treg cells (> 80% Foxp3^+^) were transferred intravenously (iv into Blimp-1-deficient mice (CKO) at 3-day post infection *T. cruzi*. The survival rate and weight loss were assessed daily.

### Isolation of leukocytes and cell culture

Briefly, the spleens of mice were homogenized and the erythrocytes lysed with 2 ml ACK lysis buffer. The liver was excised, minced with scissors, and digested with collagenase II (Sigma) for 37°C for 45 minutes. Tissue fragments were further dispersed by repeated aspiration and crushed through a 70-µm pore size nylon filter (BD Biosciences). Liver leukocytes were recovered using 40% Percoll gradient centrifugation. Erythrocytes in the cell pellets were lysed, and the remaining cells were resuspended in 5% RPMI. After processing, viability was assessed via Trypan blue exclusion, and the cell concentration determined. Leukocytes from spleen and liver were stimulated with PMA (50 ng/ml) plus ionomycin (500 ng/ml) (Sigma) and brefeldin A (Biolegend) for the analysis of intracellular cytokines by flow cytometry. Single-cell suspensions from spleen were diluted to concentration 2 x 10^6^ cell/well and dispensed into 48-well plates in a total volume of 500 ul of 5% RPMI. As a positive control, we used anti-CD3 (2ug/ml) plus anti-CD28 antibody (1 ug/ml) (BD Bioscience, USA). The minimal viability allowed was 95% and there was no significant variability between the experimental groups. The cell culture supernatants were harvested after 72 h of culture at 37 °C in 5% CO_2_, and the levels of IFN-γ and TNF-α determined by ELISA with commercial Kits (R&D Systems, USA) according to the manufacturer’s protocols.

### Flow cytometry assay

To staining the leukocytes, 1-2x10^6^ cells/tube were incubated with viability dyes (Biolegend) for 15 minutes at RT, protected from light, washed, and add the diluted antibody cocktail (100 times) for 30 minutes in PBS at 4°C. To intracellular staining, the cells were fixed with 4% paraformaldehyde for 11 minutes and permeabilized with 0.5% saponin for 30 minutes. After permeabilization, the antibodies were added and incubated for 30 minutes at 4°C. The antibodies used, obtained from BD Bioscience and Biolegend were CD45 (30-F11), CD3 (145-2C11), CD4 (RM4-5), CD8 (53-6.7), IFN-γ (XMG1.2), IL-10 (JES5-16E3), TNF-α (MP6-XT22), Foxp3 (MF-14), Granzyme B (GB11), Perforin (S16009A), CD44 (IM7), CD11b (M1/70), MHC-II (M5/114.15.2), Ly6C (HK1.4), CD11c (N418), F4/80 (T45-2342), NOS-2 (W16030C) and their respective isotype controls conjugated with different fluorochromes (FITC, PE, APC, PERCP, PE-Cy7, APC-Cy7, BV510, BV650, BV711, PE/Dazzle 594, and Pacific blue). Data acquisition and analysis were performed using the BD FACSCanto II, BD FACSymphony A1 and FlowJo software, respectively. t-SNE analysis was performed using Flow Jo T-SNE and X-Shift plugin.

### Histological analysis

To histopathological analysis, liver and heart samples were fixed in 10% buffered formalin and paraffin processed. Tissue sections of 5-µm thickness were deparaffinized and stained with hematoxylin and eosin (H&E). To identify steatosis, the liver samples were collected, frozen in OCT medium (Sakura Finetek, Inc, Torrance, CA), and tissue section of 5-µm stained with Oil Red O solution (Sigma) (19). The images of inflammatory infiltrate and lipids visualized as red-orange staining were obtained using light microscope (Leica).

### Quantitative real-time PCR (qPCR)

RNA was extracted using a Promega Kit (Promega) according to the manufacturer’s instructions. cDNA was synthesized via a reverse transcription (Kit High Capacity, Applied Biosystems). Real-time PCR for quantitative mRNA expression analyses was performed on a StepOne Plus Real-Time PCR System (Applied Biosystems) using a goTaq qPCR Master Mix fluorescence quantification system (Promega) and the primer sequences used as follows: RPL13a sense, 5’-GGAGGAGAAACGGAAGGA AAAG-3’, and antisense, 5’-TTTCCGTAACCTCAAGATCTGCTT-3’; NOS-2 sense, 5’-CGAAACGCTTCACTTCCAA-3’; and antisense, 5’-TGAGCCTATATTGCTGTGGCT-3’. Prdm1 sense, 5’- GACGGGGGTACTTCTGTTCA-3’; and antisense, 5’- GGCATTCTTGGGAACTGTGT. The standard PCR conditions were as follows: 50°C for 2 min; 95°C for 2 min; and 40 cycles of 15 seconds at 95°C, 30 seconds at 58°C and 30 seconds at 72°C, followed by a standard denaturation curve. The samples were normalized to RPL13a and the analyses performed using the cycle threshold (Ct) method, which allows for quantitative expression analysis using the formula 2^-ΔΔCt^.

### Serum TGO and TGP activity assay

To determine heart and liver damage, the activity of CK-MB (Creatine Kinase-MB), AST (Aspartate Aminotransferase) and ALT (Alanine Aminotransferase) were measured in the sera of mice non-infected and infected with *T*. *cruzi*. Quantification was performed using a specific kit (Labtest^®^) followed by read in spectrophotometer at 340 nm (EMAX Molecular Devices Corporation^®^) following the manufacturer’s recommendations.

### Cytokines measurement and nitrite quantification

Production of the cytokines IFN-γ and TNF-α was assessed in the serum and supernatant of the splenocytes culture of WT and CKO naive mice or 12 days post-infection with 10^3^ trypomastigote forms of *T*. *cruzi*. Measurements were performed through by ELISA assay, using specific kit (DuoSet^®^, R&D Systems) according to manufacturer’s instructions. The nitrite concentration was determined using the conventional Griess reaction method (20).

### Immunohistochemical and immunofluorescence microscopy

Cryopreserved liver tissues were fixed in cold acetone, washed in PBS, and the endogenous peroxidase activity blocked with 3% hydrogen peroxide or 30 minutes. Then, a Mach 1 Universal HRP Polymer Detection Kit (Biocare Medical, USA) was used according to the manufacture’s recommendations. The slides were incubated with rabbit anti-mouse NOS-2 ntibody (1:400, Santa Cruz) and counterstained with Mayer’s hematoxylin. For immunofluorescence, the slides were incubated in 0.5% saponin in PBS for 15 minutes, and nonspecific sites blocked with 1% BSA for 30 min RT. The slides were incubated with a FITC-conjugated anti-CD11c antibody and Alexa fluor 594 anti-iNOS antibody (1:100, Biolegend) overnight at 4°C. The sections were washed, and the nucleus stained and mounted with Prolong. The images were analyzed using the Leica SP5 (Leica Microsystems).

### Statistical analysis

The statistical analysis was performed using an unpaired *t*-test (Mann Whitney) or one-way ANOVA followed by Tukey’s multiple comparisons test. Survival curves were evaluated using long-rank/Mantel-Cox test. All statistical analyses were performed using Graph Pad Prism (8.0 GraphPad Software). All values were considered significantly different at P *<* 0.05.

## Results

### Blimp-1 signaling in T cells is essential for mouse resistance to *T. cruzi* infection.

**Figure 1:**
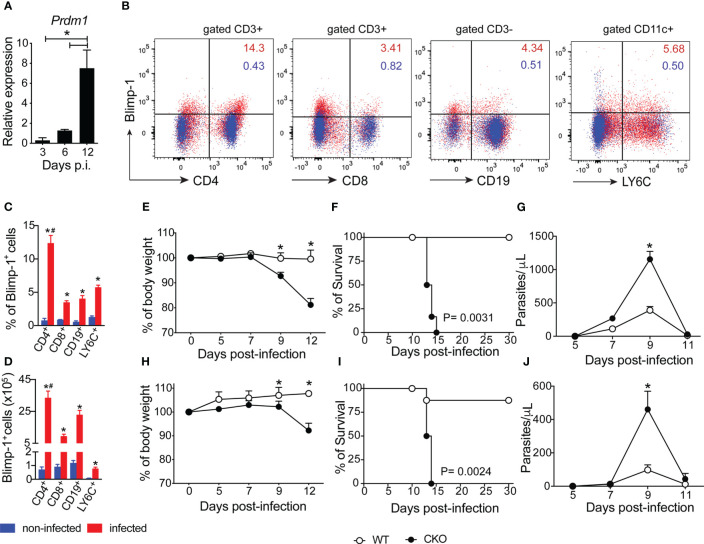
The Blimp-1 expression in T cells is essential for mouse resistance to *T*. *cruzi* infection. **(A)** Expression of Blimp-1 mRNA in spleen from WT mice indicated at days post-infection was determined by qPCR. **(B)** Representative dot plot of Blimp-1 expression in splenocytes of non-infected C57BL/6 (WT) mice and 12-day post-infection with *T. cruzi* (n=4). **(C, D)** The graph bars represent the percentage and absolute numbers of Blimp-1 expression in T cells (CD4^+^ and CD8^+^) gated on CD3^+^ and B cells (CD19^+^) gated on CD3^-^ and myeloid cells (LY6C^+^) gated on CD11c^+^CD11b^-^ by flow cytometry. **(E–H)** Body weight, **(F–I)** survival rate, and **(G–J)** blood parasitemia of Blimp-1^fl/fl^ (WT) and Blimp-1^fl/fl^ CD4^Cre^ (CKO) mice infected with 1000 **(E–G)** and 100 **(H–J)** trypomastigote forms of *T. cruzi* Y strain. Data (mean ± SEM) are representative of three experiments with five mice per group; Differences were analyzed with a one-away ANOVA using Tukey’s methods and considered significant for * ^#^ P < 0.05 (*comparison between non-infected and infected group; ^#^ comparison between CD4 T cells and CD8, CD19 and LY6C cells). Differences between the survival of experimental groups were analyzed by the log-rank (Mantel-Cox) test.

We first demonstrated that Blimp-1 expression is increased in splenocytes of mice infected with *T. cruzi* on day 12 after infection (**Figure 1A**). We further identified that CD4^+^ T cells are the primary cells expressing Blimp-1 during *T. cruzi* infection (**Figures 1B–D**). Comparing uninfected mice to infected mice, we found that Blimp-1 expression are consistently less than 2% in uninfected mice, whereas infected mice showed increased Blimp-1 expression in CD4, CD8, CD19, and LY6C positive cells. Specifically, the percentages of Blimp-1-expressing cells were 12.37% in CD4^+^ T cells, 3.49% in CD8^+^ T cells, 4.04% in CD19^+^ B cells, and 5.76% in LY6C^+^ cells (**Figures 1B–D**). To investigate the role of Blimp-1 signaling in controlling *T. cruzi*-induced disease, we infected wild-type (WT) mice and mice lacking Blimp-1 specifically in T cells (CKO mice). Unlike the WT mice, the CKO mice exhibited a significant reduction in body weight starting on day 9 post-infection (pi) and continued to worsen, regardless of whether they were infected with a low (100) or high (1000) parasite inoculum (**Figures 1E, H**). The absence of Blimp-1 in T cells led to severe weight loss, increased parasite burden, and ultimately, the death of infected mice. Additionally, while 80% of WT-infected mice survived for more than 30 days post-infection (pi), all the infected CKO mice succumbed by day 15 pi, irrespective of the size of the inoculum (**Figures 1F, I**). Furthermore, the CKO mice had significantly higher parasitemia compared to the WT mice (**Figures 1G, J**). These findings strongly indicate that Blimp-1 signaling in T cells plays a critical role in mice’s resistance to *T. cruzi* infection.

### Blimp-1 serves as a key regulator of the *T. cruzi*-induced Th1 inflammation

**Figure 2:**
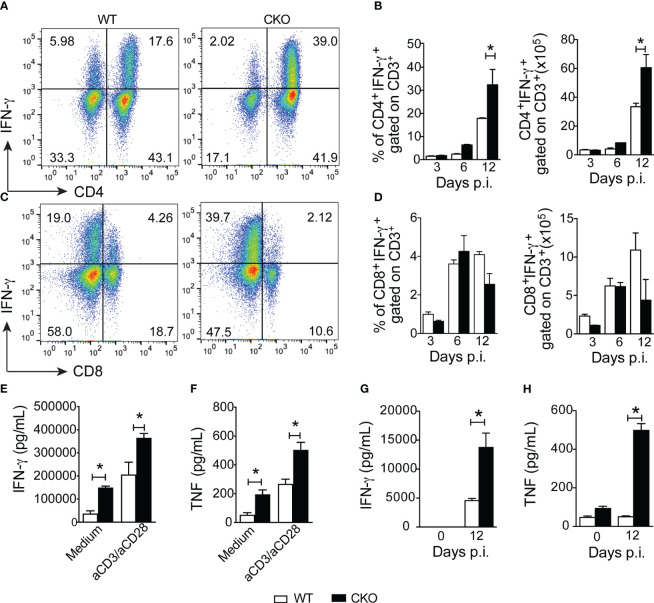
Blimp-1 arrests the Th1 cell inflammation after infection with *T*. *cruzi.* **(A, C)** Representative dot plot of CD4^+^IFN-γ^+^ and CD8^+^IFN-γ^+^ T cells from the spleen of *T. cruzi*-infection Blimp-1^fl/fl^ (WT) and Blimp-1^fl/fl^ CD4^Cre^ (CKO) mice in response to polyclonal restimulation at 12-day post-infection by cytometry flow. **(B, D)** The bar graphs show the production of IFN-γ by CD4^+^ and CD8^+^ T cells, gated on CD3, in the spleen at the indicated days post infection. **(E, G)** The levels of IFN-γ and TNF **(F, H)** in supernatants of splenocytes, stimulated or not for 72 hours with anti-CD3 plus CD28 and sera, respectively, of *T. cruzi*-infected WT and CKO mice at 12 dpi are shown. Data (mean ± SEM) are representative of two experiments with five mice per group. Differences were analyzed with a one-way ANOVA using Tukey’s method and considered significant for * p< 0.05.

To understand how Blimp-1 contributes to resistance against the infection, we investigated whether its deletion in T cells affects the Th1 response in infected mice. Notably, at 12 days post-infection, we observed a higher frequency and greater number of CD4^+^ T cells producing IFN-γ in the spleen of CKO mice compared to WT mice (**Figures 2A, B**). However, the frequency and numbers of CD8^+^ T cells producing IFN-γ were similar in both groups (**Figures 2C, D**). Furthermore, Blimp-1-deficient mice displayed significantly elevated levels of the proinflammatory cytokines IFN-γ and TNF in the supernatant of splenocytes restimulated or not (**Figures 2E, F**) and in their serum (**Figures 2G, H**) compared to WT mice.

**Figure 3:**
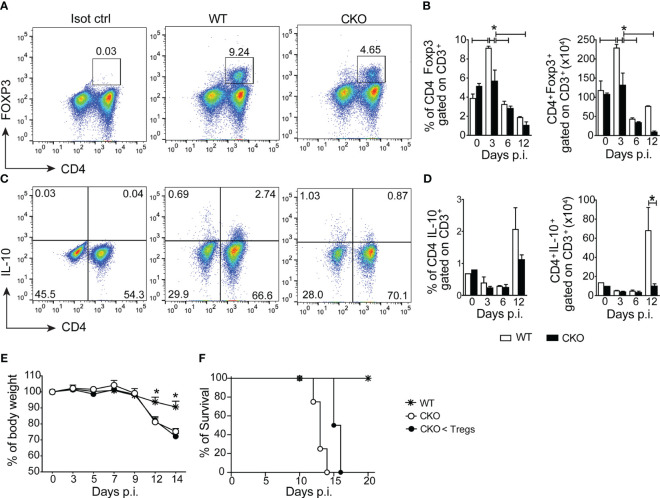
The deletion of Blimp-1 in T cells abrogates the of regulatory T cells and IL-10 production in the infected mice. **(A)** Representative dot plot of CD4^+^Foxp3^+^ T cells from spleen of *T. cruzi*-infection Blimp-1^fl/fl^ (WT) and Blimp-1^fl/fl^ CD4^Cre^ (CKO) mice at 3 dpi. **(B)** The graph bars represent the percentage and absolute numbers of CD4^+^Foxp3^+^ T cells from the spleen at the indicated days post-infection. **(C)** Representative dot plot of IL-10 production by CD4^+^ T cells from the spleen of *T. cruzi*-infection WT and CKO mice in response to polyclonal restimulation at 12 dpi. **(D)** The graph bars represent the percentage and absolute numbers of IL-10-producing CD4^+^ T cells in the spleen at the indicated days post-infection. **(E)** Body weight, and **(F)** survival rate of WT and CKO mice infected with 100 trypomastigote forms of *T. cruzi* Y strain, either with transferred Tregs or not at day 3 post-infection. Data (mean ± SEM) are representative of two experiments with five mice per group. Differences were analyzed with a one-way ANOVA using Tukey’s method and considered significant for *p< 0.05. Differences between the survival of experimental groups were analyzed by the log-rank (Mantel-Cox) test.

During the early acute phase of infection (3 days post-infection), there was a reduction in the frequency and number of CD4^+^Foxp3^+^ Tregs in the spleen of CKO mice compared to WT mice (**Figures 3A, B**). However, this difference was not observed at 6- and 12-days post-infection. Additionally, at 12 days post-infection, the spleen of CKO mice showed a decrease in the number of CD4^+^ T cells producing IL-10 compared to WT mice (**Figures 3C, D**).

Despite these observations, even when we performed an adoptive transfer of Tregs from WT mice to CKO mice, the infected recipients did not experience protection against weight loss (**Figure 3E**) and death (**Figure 3F**). This indicates that the increased susceptibility to the infection and exacerbated inflammatory response in CKO mice is independent of Tregs. In summary, the absence of Blimp-1 in T cells leads to enhanced Th1 inflammation during *T. cruzi* infection. Moreover, the increased susceptibility to the infection and the exacerbated inflammatory response are not solely reliant on Tregs, suggesting that Blimp-1 regulates other aspects of the immune response in the context of Chagas disease.

### Blimp-1 plays a protective role against hepatic damage and dysfunction caused by *T. cruzi.*

**Figure 4:**
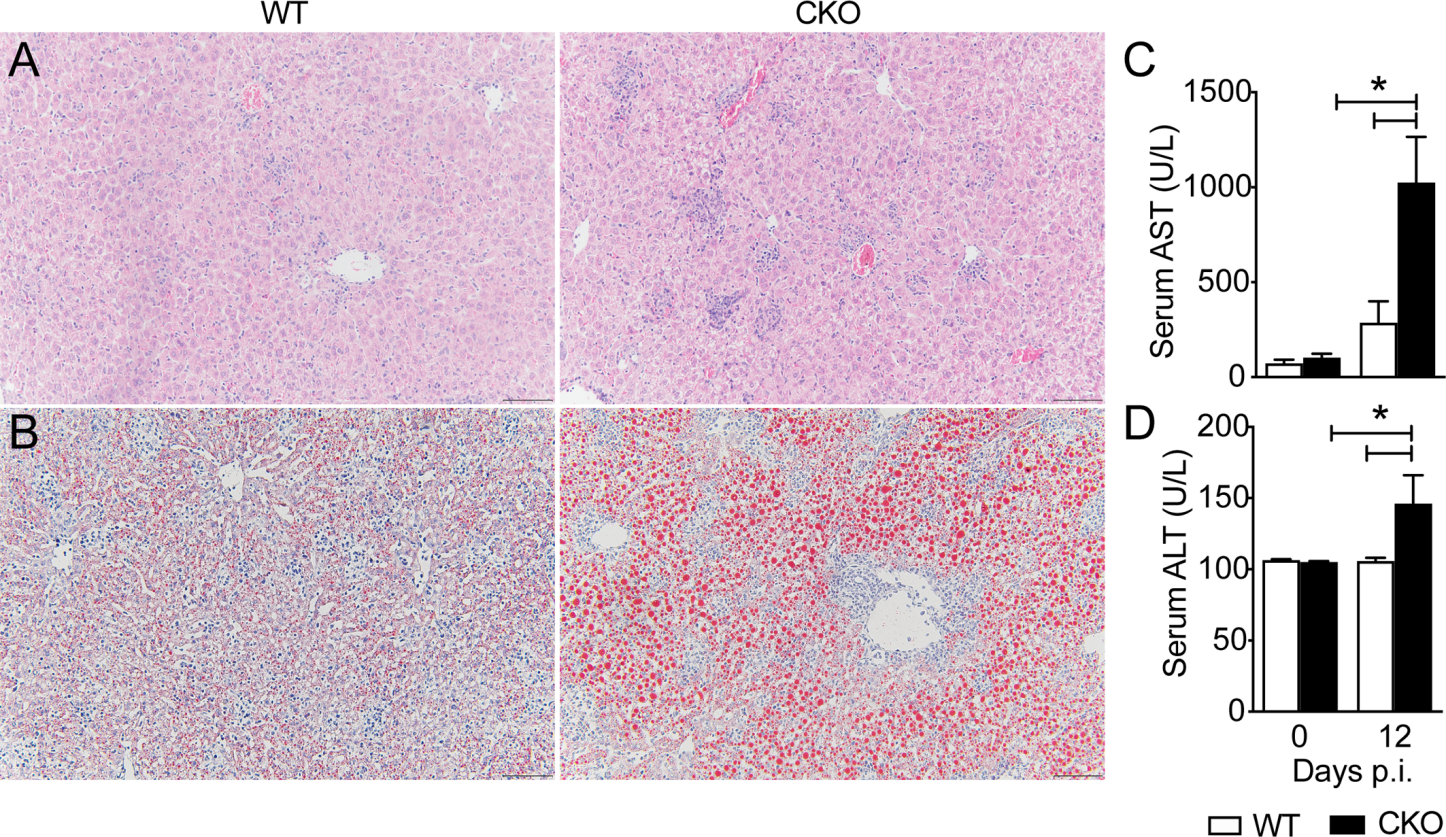
Blimp-1 is important for the liver function of *T. cruzi*-infected mice. Representative images from **(A)** H&E and **(B)** Oil Red O staining of liver tissue from Blimp-1^fl/fl^ (WT) and Blimp-1^fl/fl^ CD4^Cre^ (CKO) mice 12-day post-infection with *T. cruzi* Y strain. Scale bars = 50 μm. **(C)** AST and **(D)** ALT levels in the sera of *T. cruzi* –infected at 12 dpi. Data (mean ± SEM) are representative of two experiments with four mice per group. Differences were analyzed with a one-way ANOVA using Tukey’s method and considered significant for * p< 0.05.

**Figure 5:**
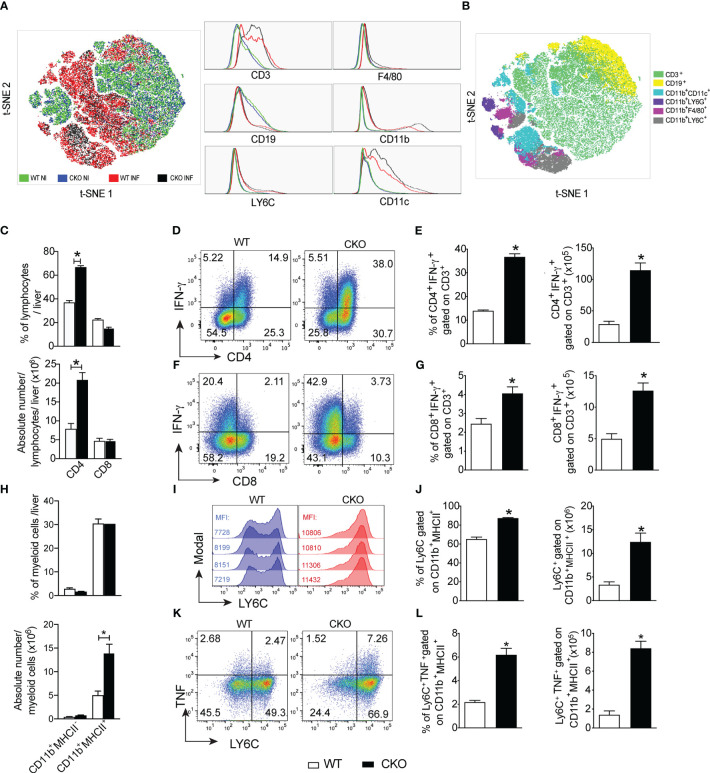
The deletion of Blimp-1 in T cells favors a liver Th1 inflammation and contributes to local monocyte activation. **(A)** The t-SNE plot of leukocytes isolated from the liver of Blimp-1^fl/fl^ (WT) and Blimp-1^fl/fl^ CD4^Cre^ (CKO) mice naïve (NI) and infected (INF) with *T. cruzi* Y strain at 12 dpi. **(B)** t-SNE plot showing all clusters and subpopulations determined by the surface markers CD3, CD19, CD11b, CD11c, F4/80, Ly6G, and LY6C obtained with X-shift plugins and flow cytometry data. **(C)** Percentage and absolute number of lymphocytes CD4^+^ and CD8^+^ T cells gated on CD3^+^. **(D, F)** Representative dot plots and **(E, G)** bar graphs showing percentage and absolute number of IFN-γ production by CD4^+^ and CD8^+^ T cells gated on CD3 from the liver in response to polyclonal restimulation at 12 dpi. **(H)** Percentage and absolute number of myeloid cells CD11b^+^MHC-II^-^ and CD11b^+^MHCII^+^. **(I)** Representative histograms of CD11b^+^MHCII^+^ cells from liver stained for LY6C; MFI (mean fluorescence intensity). **(J)** Percentage and absolute numbers of LY6C-expressing CD11b^+^MHCII^+^ cells from *T. cruzi*-infected mice. **(K)** Representative dot plot and **(L)** bar graph showing the percentage and absolute number of LY6C^+^CD11b^+^MHCII^+^ cells expressing TNF from the liver at 12 dpi. Data (mean ± SEM) are representative of two experiments with four mice per group. Differences were analyzed with a one-way ANOVA using Tukey’s or unpaired *t*-test methods (Mann Whitney) and considered significant for * p< 0.05.

The histological analysis of heart and liver revealed a huge increase in leukocyte migration to the liver (**Figure 4A**), but not heart (**Supplementary Figure 1A**) of CKO infected mice on day 12 pi, with extensive hepatocyte vacuolization and stained lipid droplets (**Figure 4B**), indicative of steatosis. In accordance, the activity of AST (**Figure 4C**) and ALT (**Figure 4D**) were significantly increased in *T. cruzi*-infected CKO mice compared to WT mice, suggesting a protective role mediated by Blimp-1 signaling. In contrast, we did not find significant inflammatory infiltrate in cardiac tissue of CKO and WT mice at day 12 pi (**Supplementary Figure 1A**) that was confirmed by the similar activity of CKMB among the groups (**Supplementary Figure 1B**). In fact, the flow cytometry analysis showed a dramatic increase in the leukocytes infiltrated into the liver of infected WT and CKO compared to uninfected mice (**Figure 5A**). Still, liver infiltrated leukocytes from infected WT and CKO mice exhibit a high CD3^+^, CD11b^+^, CD11c^+^, F4/80^+^, and LY6C^+^ expression compared to uninfected (**Figure 5A**). We found 6 clusters of B and T lymphocytes (CD19^+^ and CD3^+^) and myeloid cells (CD11b^+^CD11c^+^, CD11b^+^LY6C^+^, CD11b^+^LY6G^+^, and CD11b^+^F4/80^+^) in the liver from WT and CKO infected compared to uninfected mice (**Figure 5B**). Both, the frequency and number of CD4^+^ (**Figure 5C**), but not CD8^+^ (**Figure 5C**), T lymphocytes infiltrating the liver of CKO mice are increased compared to WT mice at 12 dpi. Moreover, in the absence of Blimp-1, the frequency, and numbers of CD4^+^ and CD8^+^ T cells producing IFN-γ were increased compared to WT mice (**Figures 5D–G**). Therefore, Blimp-1 expression is essential to control a *T. cruzi*-induced liver Th1 inflammation. Additionally, in the liver tissue of Blimp-1 deficient animals, there is an increase in Th1 lymphocytes along with a greater recruitment of activated myeloid cells, specifically inflammatory monocytes (CD11b^+^MHCII^+^LY6C^+^), as indicated by the higher expression of LY6C in the population of CD11b^+^MHCII^+^ cells from CKO mice compared to WT mice (**Figures 5H–J**). Furthermore, we found an elevated frequency and number of TNF-producing monocytes in the liver of CKO mice compared to WT mice (**Figures 5K, L**), suggesting their activation and functionality.

To further investigate the impact of the high leukocyte infiltration in the liver of Blimp-1-deficient animals, we treated them with dexamethasone (Dexa). Although there were no changes in the numbers of circulating parasites in the blood (**Supplementary Figure 2A**), we observed a reduction in weight loss (**Supplementary Figure 2B**) and mortality (**Supplementary Figure 2C**) in Dexa-treated CKO mice compared to untreated CKO mice, indicating partial protection against *T. cruzi* infection with glucocorticoid treatment. These findings suggest that Blimp-1 plays a role in controlling liver Th1 inflammation induced by *T. cruzi* infection and contributes to the activation of local monocytes. However, it is important to note that susceptibility to *T. cruzi* infection involves other factors beyond inflammation, as evidenced by the partial protection provided by blocking the inflammation in Blimp-1-deficient mice.

### Blimp-1 signaling in T cells reduces *T. cruzi* infection

**Figure 6:**
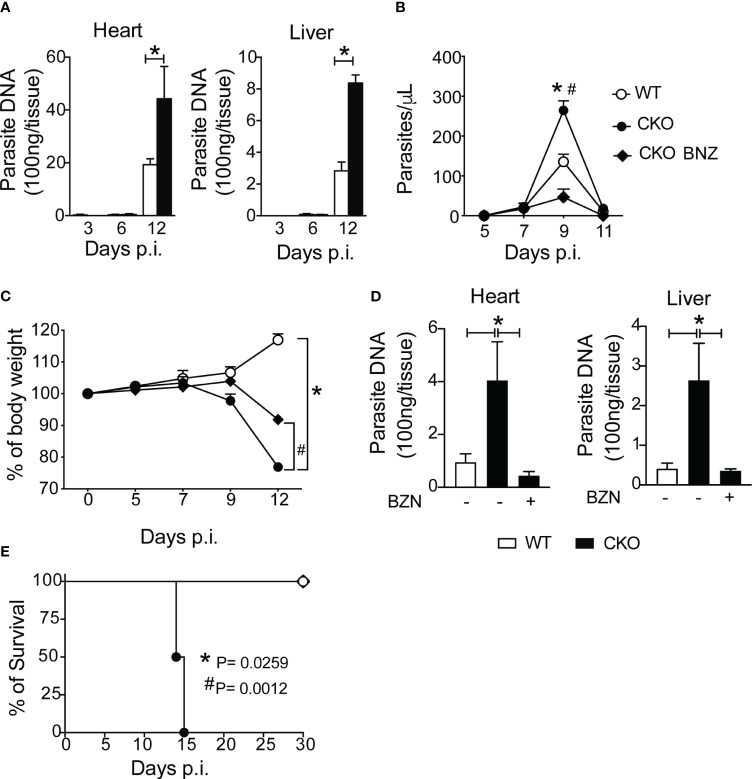
Blimp-1 controls the parasitism after infection with *T*. *cruzi.* **(A)** Quantitative PCR analysis of *T. cruzi* DNA isolated from heart and liver from Blimp-1^fl/fl^ (WT) and Blimp-1^fl/fl^ CD4^Cre^ (CKO) mice infected with 1000 trypomastigote forms of *T. cruzi* Y strain. **(B)** Blood parasitemia, **(C)** body weight, **(D)** quantitative PCR analysis of *T. cruzi* DNA isolated from heart and liver, and **(E)** survival rate of WT and CKO mice infected with 100 trypomastigote forms of *T. cruzi* Y strain and treated at day 7 and 9 post-infection with 100 mg/Kg of Benznidazole (BNZ) or vehicle. Data (mean ± SEM) are representative of two experiments with five mice per group. Differences were analyzed with a one-way ANOVA using Tukey’s method and considered significant for * p< 0.05. Differences between the survival of experimental groups were analyzed by the log-rank (Mantel-Cox) test.

In terms of parasitism control, CKO mice displayed a high number of circulating parasites compared to WT mice, and at 12 dpi, CKO mice exhibited an augmented amount of parasite DNA in the heart and liver tissues compared to infected WT mice (**Figure 6A**). Treatment with benznidazole, the standard trypanocidal drug (21) for *T. cruzi* infection, resulted in reduced circulating parasites, percentage of body weight, and parasite DNA in the heart and liver at 12 dpi, thereby protecting CKO mice from death compared to vehicle-treated mice (**Figures 6B–E**). These data demonstrate that the absence of Blimp-1 in T cells leads to enhanced parasite persistence during *T. cruzi* infection. Additionally, control of parasitism primarily involves infiltrating CD8^+^ T cells, nitric oxide (NO) production, and antibody production.

**Figure 7:**
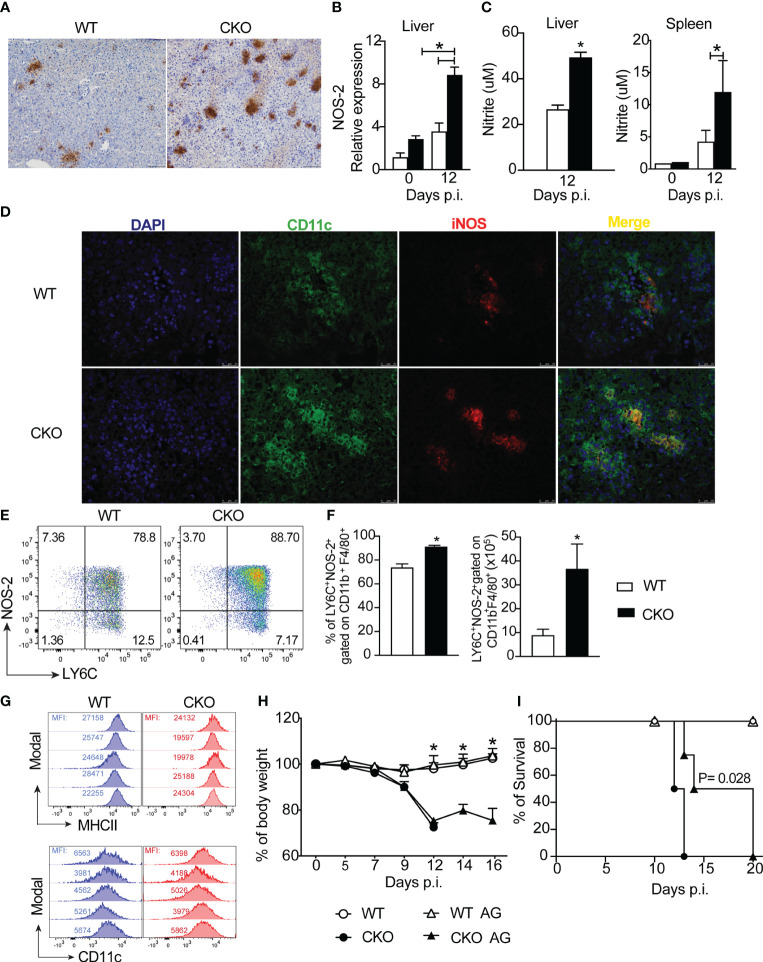
The deletion of Blimp-1 in T cells contributes to a high NO production by MO-DCs cells. **(A)** Representative images from immunohistochemistry staining for iNOS and **(B)** iNOS mRNA expression of liver tissue from Blimp-1^fl/fl^ (WT) and Blimp-1^fl/fl^ CD4^Cre^ (CKO) mice at 12-day post-infection with *T. cruzi* Y strain. Scale bars = 50 μm. **(C)** Nitrite quantification by Griess reaction in the supernatant of WT and CKO leukocytes from liver and splenocytes cultived by 24 hours. **(D)** Representative images from immunofluorescent staining for iNOS and CD11c of liver tissue from WT and CKO mice. **(E)** Representative dot plot and **(F)** percentage and absolute numbers of LY6C^+^NOS-2^+^-expressing CD11b^+^F4/80^+^ cells from liver at 12 dpi from *T. cruzi*-infected mice. **(G)** Representative histograms of LY6C^+^NOS-2^+^ cells gated on CD11b^+^F4/80^+^ cells from the liver stained for MHCII and CD11c; MFI (mean fluorescence intensity). **(H)** Body weight and **(I)** survival rate of WT and CKO mice infected with 100 trypomastigote forms of *T. cruzi* Y strain and treated for 7 days daily starting on day 7 post-infection with 50 ug/mice of Aminoguanidine (AG) or vehicle. Data (mean ± SEM) are representative of two experiments with four mice per group. Differences were analyzed with a one-way ANOVA using Tukey’s method or unpaired *t*-test (Mann Whitney) and considered significant for * p< 0.05. Differences between the survival of experimental groups were analyzed by the log-rank (Mantel-Cox) test.

Moving forward, we further examined the expression of nitric oxide synthase (iNOS), an enzyme involved in nitric oxide production, in the liver tissue of infected mice. We observed heightened iNOS protein and gene expression in the liver tissue of infected CKO mice compared to WT mice (**Figures 7A, B**). This finding was corroborated by increased nitrite production in leukocytes isolated from the liver and spleen of CKO mice compared to WT mice (**Figure 7C**). Immunofluorescence data revealed elevated iNOS staining primarily in myeloid cells, specifically CD11c^+^ cells, in the hepatic tissue of Blimp-1 deficient mice compared to WT mice (**Figure 7D**).

Next, we sought to identify which cell subpopulation in the liver, induced by *T. cruzi* infection, was responsible for NO production. Our analysis revealed a higher frequency and absolute number of LY6C^+^NOS-2^+^ cells, gated on CD11b^+^F4/80^+^ cells (as shown in the strategy gating in **Supplementary Figure 3A**) in the liver tissue of CKO mice compared to WT mice (**Figures 7E, F**). Importantly, these cells exhibited high expression of MHCII and CD11c (**Figure 7G**), suggesting that the primary source of NO production was monocyte-derived dendritic cells (MO-DCs) infiltrating the hepatic tissue in response to *T. cruzi* infection.

Given the elevated production of NO in the liver tissue of Blimp-1 deficient mice, we investigated whether blocking NOS with aminoguanidine (AG) could prevent acute mortality induced by *T. cruzi* infection in CKO mice. While the treatment with AG did not prevent weight loss (**Figure 7H**), it did partially prolong the survival of AG-treated CKO mice compared to untreated mice (**Figure 7I**).

Next, we found that the production of anti-*T. cruzi* total IgG antibodies in the serum were similar between CKO and WT mice (**Supplementary Figure 4A**).

**Figure 8:**
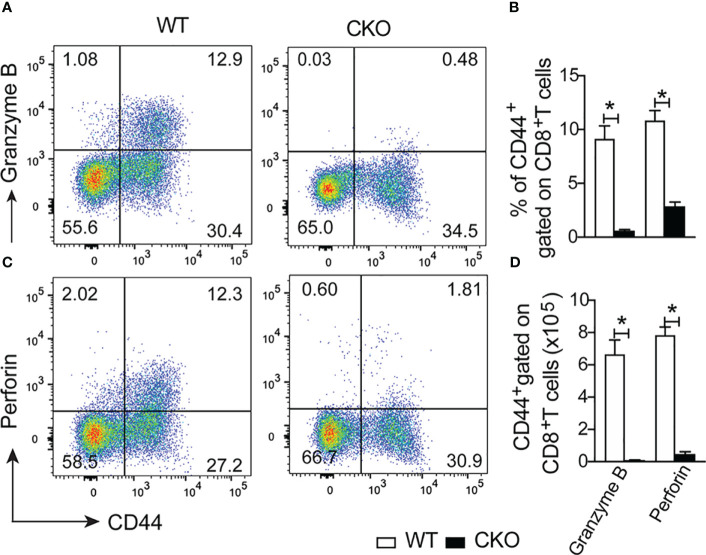
Lack of the Blimp-1 in T cells leads to dysfunction of cytotoxic CD8+ T cells in the acute *T. cruzi* infection. **(A, C)** Representative dot plot of granzymeB^+^CD44^+^ and perforin^+^CD44^+^ gated on CD8^+^ T cells from the spleen of *T. cruzi*-infection Blimp-1^fl/fl^ (WT) and Blimp-1^fl/fl^ CD4^Cre^ (CKO) mice in response to polyclonal restimulation at 12-day post-infection by cytometry flow. **(B, D)** The bar graphs show the expression of granzyme B and perforin in CD44^+^ cells, gated on CD8^+^ T cells, in the spleen at 12 days post-infection. Data (mean ± SEM) n= 5 mice per group. Differences were analyzed with a one-way ANOVA using Tukey’s method and considered significant for * p< 0.05.

Finally, we assess whether the deletion of Blimp-1 in T cells would compromise the cytotoxic function of CD8^+^ T cells. Notably, at 12 days post-infection, we found a reduction of the frequency and number of CD8^+^ T cells activated (CD44^+^) producing granzyme B (**Figures 8A, B**) and perforin (**Figures 8C, D**) in the spleen of CKO mice compared to WT mice. These results suggest that the failure to control parasites in the absence of Blimp-1 is not due to a lack of antibody and nitric oxide production, but due the reduction of the cytotoxic CD8^+^ T cells. In conclusion, our findings strongly indicate that Blimp-1 plays a critical role in control the parasite, dependent on cytotoxic CD8^+^ T cells, and its absence leads to an uncontrolled acute inflammatory process, ultimately resulting in high susceptibility to *T. cruzi* infection. Additionally, the enhanced production of nitric oxide by monocyte-derived dendritic cells contributes to the exacerbation of the inflammatory response and the pathogenesis of Chagas disease in Blimp-1 deficient mice.

## Discussion

Understanding the mechanisms that determine host resistance or susceptibility to *T. cruzi* infection is crucial for identifying new targets to prevent severe forms of Chagas disease. In this context, we investigated the role of Blimp-1 signaling in T cells during *T. cruzi* infection. We initially expected that Blimp-1 deficiency in T cells might confer increased resistance to the infection, as observed in other infectious diseases, such as *Plasmodium chabaudi* infection (16). However, our findings were unexpected, as *T. cruzi*-infected mice lacking Blimp-1 in T cells displayed 100% mortality during the acute phase, regardless of the parasite inoculum size.

Blimp-1 is known to play crucial roles in regulating B- and T-lymphocyte functions, including Th1, Th17, and cytotoxic T cells, which are all important for controlling Chagas disease (12, 15, 22, 23). The increased susceptibility of CKO mice to *T. cruzi* infection warranted further investigation, especially considering the involvement of Blimp-1 in suppressing the immune response in infectious diseases through IL-10 production (16).

Our data clearly indicated that Blimp-1 signaling in T cells plays an essential role in limiting tissue parasitism, thus preventing the development of uncontrolled inflammation, hepatic damage, dysfunction, and ultimately the death of the mice during *T. cruzi* infection. This contrasts with findings in other inflammatory disease models, where Blimp-1 deletion in T cells led to exacerbated cytokine production and worsened inflammation (14, 15). In *Plasmodium chabaudi* and *Plasmodium yoelii* infections, Blimp-1 in T cells mediated susceptibility through Tr1 cell-derived IL-10, which prevented tissue damage caused by TNF and contributed to parasite replication (24).

Chagas disease has been associated with elevated levels of local and systemic pro-inflammatory cytokines (8), which could induce Blimp-1 expression in T cells. Once expressed, Blimp-1 controls the expression of multiple transcription factors, including T-box–containing protein, IRF-4, and B-cell lymphoma 6, which are required for the functions of Th1, Treg, and follicular helper T cells, respectively (23, 25, 26). The infection with *T. cruzi* in Blimp-1-deficient mice triggered a robust pro-inflammatory response, evidenced by high levels of cytokines such as TNF and IFN-γ, both locally and systemically on day 12 post-infection, but not earlier. Additionally, the activation of NOS-2 was observed, mainly produced by inflammatory monocytes highly present at the site of *T. cruzi* infection.

Inflammatory monocytes originate from the bone marrow and migrate to the site of infection during inflammation. Upon recruitment, these monocytes can differentiate into dendritic cells or macrophages or preserve their monocyte phenotype (27, 28). Their functions can vary depending on the tissue environment (29). In experimental *Leishmania donovani* infection, Th1 cells drive monocyte activation in the bone marrow and contribute to parasite control (30). However, despite the accumulation of monocytes in CKO mice and the differentiation of MO-DCs cells and NO production, parasite replication remained uncontrolled.

Moreover, Blimp-1 is involved with activated of cytotoxic CD8^+^ T cells in the *T. cruzi* infection. Lack of Blimp-1 in T cells, leads to a failure in the expression of granzyme B and perforin in CD8^+^ T cells, what could explain the persistence of the parasite. CD8^+^ T cells play a critical role in acute resistance to experimental *T. cruzi* infection (31). The differentiation, expansion, and activating of *T. cruzi*-specific CD8^+^ cytotoxic T cells are dependent on parasite multiplication and CD4^+^ T cells (32). In influenza virus infection, Blimp-1 controls the differentiation of cytotoxic effector cells and memory CD8 T cells (22). Therefore, failure of the CD8^+^ T cells response in Blimp-1 deficient mice leads to the persistence of parasites in the tissues during the acute phase promotes leukocyte recruitment and the production of inflammatory cytokines, which is detrimental to the host.

Although the mechanisms involved in parasite replication restriction, such as IFN-γ and NO, were induced in T cell-specific Blimp-1 deficient mice infected with *T. cruzi*, the abundant production of mediators may lead to tissue damage and dysfunction (33, 34) and ultimately result in animal death. Interestingly, treatment with a low dose of glucocorticoids, to prevent inflammation, in Blimp-1-deficient mice resulted in increased survival. Moreover, inhibiting NO production, by treatment with AG, known to suppress the immune response (33), also led to increased mice survival. These findings suggest that Blimp-1 expression in T cells negatively modulates the production of TNF, IFN-γ, and NO, preventing tissue damage during the infection.

Therefore, the generation of regulatory mechanisms mediated by Blimp-1 is crucial for maintaining homeostasis. The deletion of Blimp-1 in T cells leads to a reduction of CD4^+^Foxp3^+^ T cells during the acute phase of infection and significantly decreased IL-10 production on day 12 post-infection, which could explain the observed increased inflammation. The lack of Blimp-1 in T cells results in increased proliferation, IL-2, and IFN-γ production and IL-10 decreased after T cell stimulation (35). On the contrary, there is a negative feedback regulatory loop in activated T cells, that IL-2 inhibits its own production through induction of Blimp-1 while promoting an effector cell phenotype (36). In fact, reduction of Tregs during *T. cruzi* infection could be due a massive accumulation of effector immune cells. Moreover, the expression of specific T-effector cell molecules on Tregs can limits its ability to ameliorate pathology (37, 38). Also, Blimp-1 expression in Tregs modulates their expansion, function, and stability contributing to its role in homeostasis (26, 39). The IL-10 production is dependent of Blimp-1 and represses white adipose tissue (40). Moreover, Blimp-1 in T cells controls Th1 and Th17 cells in autoimmune diseases.

Our findings also revealed that the deletion of Blimp-1 in T cells during acute *T. cruzi* infection leads to lipid accumulation in the liver tissue, indicative of steatosis. The accumulation of Th1 cells, dendritic cells, inflammatory monocytes, TNF, IFN-γ, and NO, significantly increased in infected CKO compared with WT mice, could be responsible for the observed non-alcoholic steatosis. In fact, the inflammation induced by *T. cruzi* infection is a potent risk factor for non-alcoholic steatohepatitis (41). As hepatocytes form one of the first lines of defense against the parasite (42) and play a role in regulating the inflammatory response (43), their remarkable lesion in CKO mice certainly affects parasite elimination. The decreased clearance and destruction of blood trypomastigotes (44) and the damage signals generated during acute infection (43, 45) should affect liver functionality. In fact, the liver, as a metabolic regulator of lipids, carbohydrates, and proteins, possesses specific immunological properties and is home to numerous resident and non-resident cells involved in the regulation of inflammatory and immune responses (42, 46).

We propose that in the absence of Blimp-1 in T cells, the persistence of parasites leads to hepatic infiltration and the release of many inflammatory cytokines that participate in the early defense response, contributing to additional liver damage. Treatment with benznidazole reduces circulating parasites and parasite burden in the tissues, thus protecting the mice from death, indicating that susceptibility to *T. cruzi* infection is associated with the inability to control parasite replication and not a possible autoimmune response.

In summary, our study highlights the crucial role of Blimp-1 signaling in T cells for host resistance to *T. cruzi* infection. Blimp-1 expression in T cells is essential for restricting parasite replication via induction of cytotoxic CD8^+^ T cells response and Th1 inflammation at the site of infection, preventing the recruitment and activation of inflammatory monocytes and the subsequent release of mediators such as TNF and NO, which cause hepatic damage and dysfunction. Importantly, the activation of the Blimp-1 pathway is necessary for host and parasite survival. Overall, our findings provide insight into potential therapeutic targets for the control of acute inflammatory diseases and contribute to a better understanding of the molecular mechanisms underlying the pathophysiology of Chagas disease.

## Data availability statement

The original contributions presented in the study are included in the article/**Supplementary Material**. Further inquiries can be directed to the corresponding author.

## Ethics statement

The animal study was approved by Ethics Committee at the University of São Paulo (CEUA) Brazil (123/2017). The study was conducted in accordance with the local legislation and institutional requirements.

## Author contributions

LB: Conceptualization, Formal Analysis, Investigation, Methodology, Writing – original draft, Writing – review & editing. LS: Methodology, Writing – review & editing. FP: Methodology, Writing – review & editing. GB: Formal Analysis, Methodology, Writing – review & editing. VC: Methodology, Writing – review & editing. JS: Conceptualization, Funding acquisition, Investigation, Project administration, Resources, Supervision, Writing – original draft, Writing – review & editing.

## Supplementary material

Supplementary Figure 1 Deletion of Blimp1 in T cells did not affect cardiac tissue in the acute phase of the T. cruzi infection. **(A)** Representative images from H&E staining of heart tissue from WT and CKO mice at 12-day after infection with 1000 trypomastigote forms of T. cruzi Y strain. Scale bar = 100 μm. **(B)** Serum CK-MB level of WT and CKO mice non-infected mice and 12-day post-infection. Data (mean ± SEM) are representative of two experiments with four experiments per group. Differences were analyzed with a one-way ANOVA using Tukey’s method and considered significant for *p< 0.05.

Supplementary Figure 2 Blocked of inflammation partially control the susceptibility T cruzi infection in T cell Blimp-1-deficient mice. **(A)** Blood parasitemia, **(B)** body weight, and **(C)** survival rate of WT and CKO mice infected with 100 trypomastigote forms of T. cruzi Y strain and treated at day 9, 12 and 15 post-infection with 1 mg/Kg of Dexamethasone (Dexa) or vehicle. Data (mean ± SEM) are representative of two experiments with four experiments per group. Differences were analyzed with a oneway ANOVA using Tukey’s method and considered significant for *p< 0.05. Differences between the survival of experimental groups were analyzed by the log-rank (Mantel-Cox) test.

Supplementary Figure 3 Gate strategy for identifying and phenotyping NOS-2+ LY6C+ inflammatory monocyte cells in the liver of T. cruzi-infected mice. **(A)** The gating strategy commences with the isolation of single cells (G1). A standardized gating procedure is then applied, successively selecting CD45+Live/Dead- viable leukocytes (G2). To analyze myeloid cells based on their size (FSC) and granularity (SSC), gating is performed in G3. For the examination of inflammatory monocytes, CD11b+F4/80+ cells are gated in G4. Subsequent markers (LY6C and NOS-2) are identified as depicted in the dot plot.

Supplementary Figure 4 Deletion of Blimp-1 in T cells did not change the anti-T. cruzi antibodies production during T. cruzi infection. **(A)** Serum anti-T.cruzi antibody level of WT and CKO mice non-infected mice and 12-day after infection with 1000 trypomastigote forms of T. cruzi Y strain. Data (mean ± SEM) are representative of two experiments with four experiments per group. Differences were analyzed with a oneway ANOVA using Tukey’s method and considered significant for *p< 0.05.
