# Supplementary figures and images for: Blimp-1 signaling pathways in T lymphocytes is essential to control the Trypanosoma cruzi infection-induced inflammation
Source: Front Immunol. 2023 Oct 16;14:1268196. doi: 10.3389/fimmu.2023.1268196 (PMC10614018; doi:10.3389/fimmu.2023.1268196)

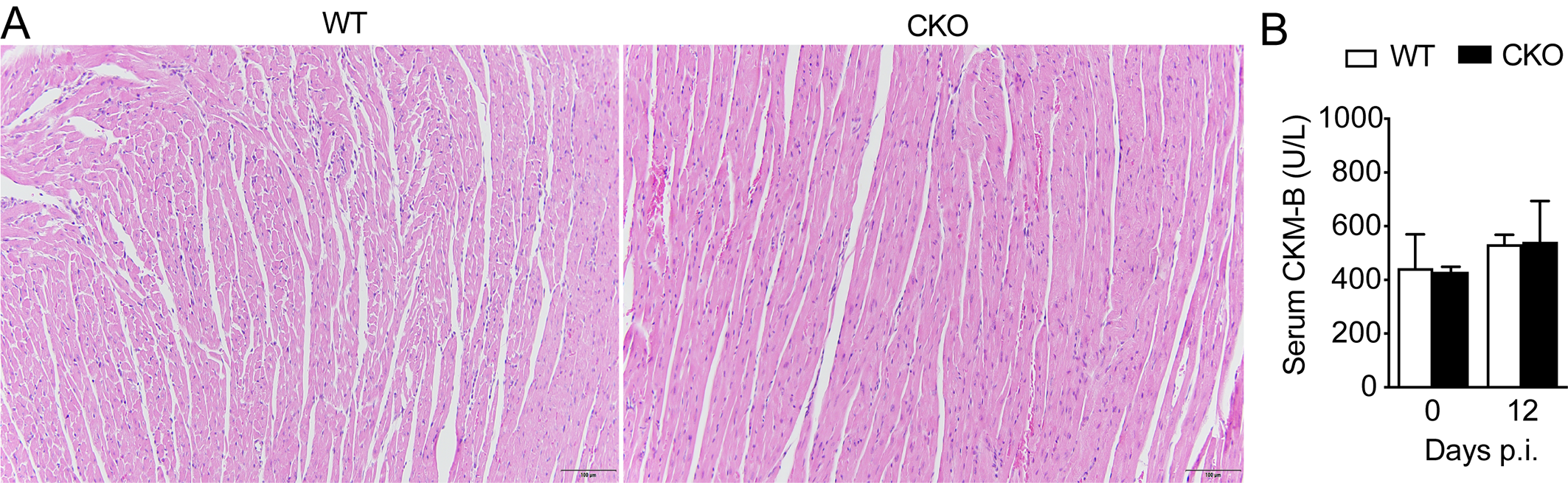

Supplement: Supplementary Figure 1 — Deletion of Blimp1 in T cells did not affect cardiac tissue in the acute phase of the T. cruzi infection. (A) Representative images from H&E staining of heart tissue from WT and CKO mice at 12-day after infection with 1000 trypomastigote forms of T. cruzi Y strain. Scale bar = 100 μm. (B) Serum CK-MB level of WT and CKO mice non-infected mice and 12-day post-infection. Data (mean ± SEM) are representative of two experiments with four experiments per group. Differences were analyzed with a one-way ANOVA using Tukey’s method and considered significant for *p< 0.05. [file Image_1.tif]

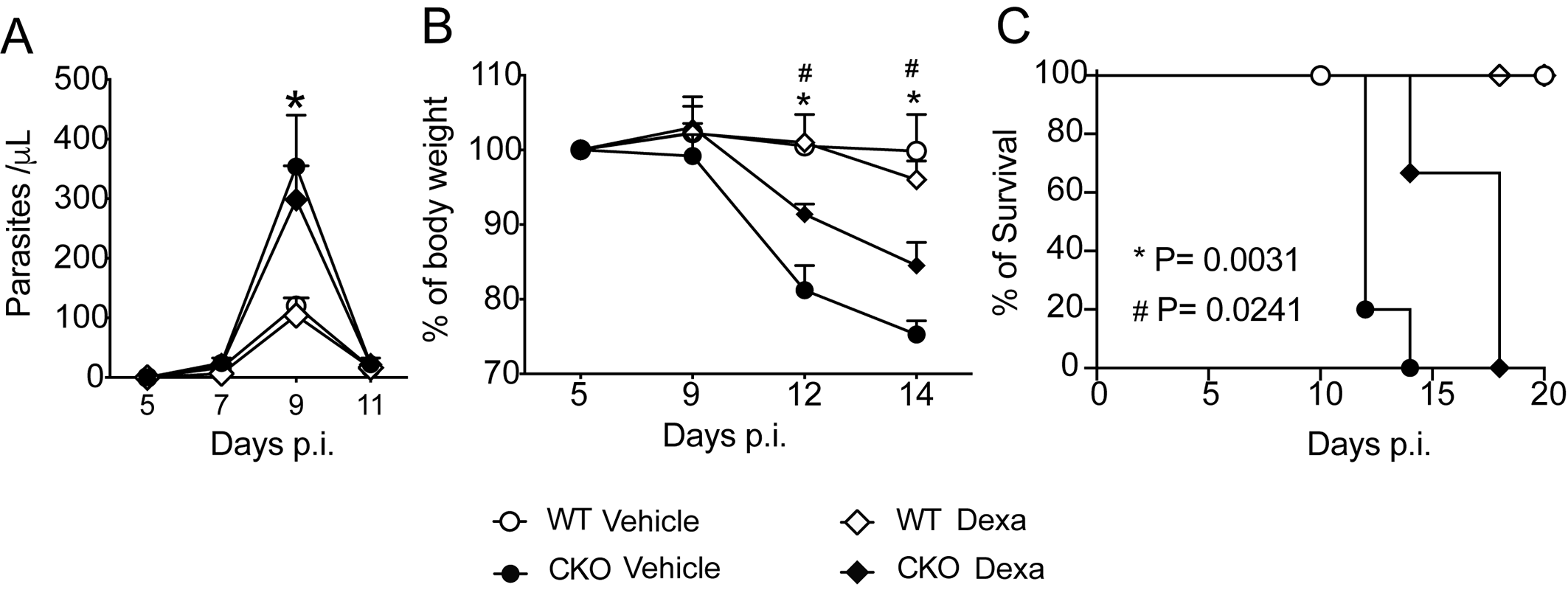

Supplement: Supplementary Figure 2 — Blocked of inflammation partially control the susceptibility T cruzi infection in T cell Blimp-1-deficient mice. (A) Blood parasitemia, (B) body weight, and (C) survival rate of WT and CKO mice infected with 100 trypomastigote forms of T. cruzi Y strain and treated at day 9, 12 and 15 post-infection with 1 mg/Kg of Dexamethasone (Dexa) or vehicle. Data (mean ± SEM) are representative of two experiments with four experiments per group. Differences were analyzed with a oneway ANOVA using Tukey’s method and considered significant for *p< 0.05. Differences between the survival of experimental groups were analyzed by the log-rank (Mantel-Cox) test. [file Image_2.tif]

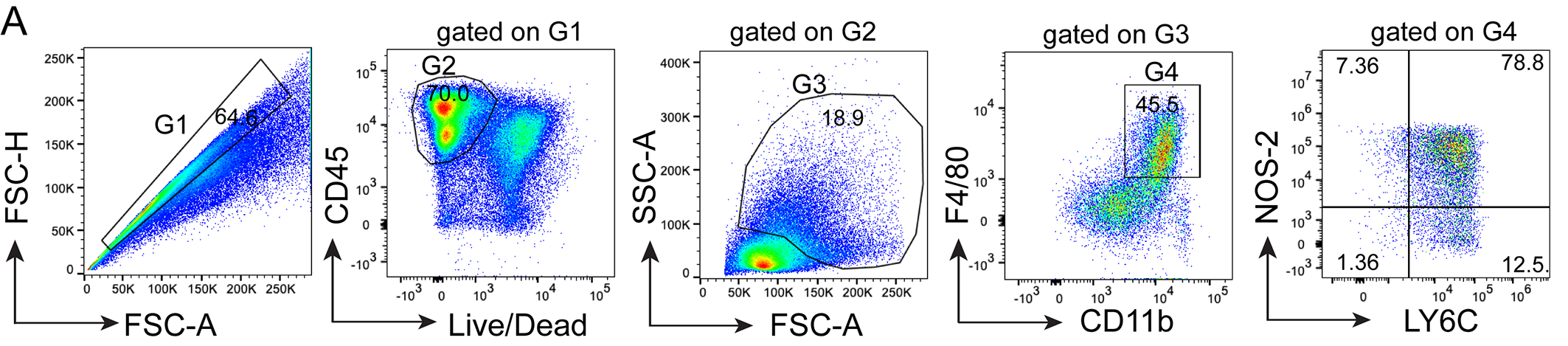

Supplement: Supplementary Figure 3 — Gate strategy for identifying and phenotyping NOS-2+ LY6C+ inflammatory monocyte cells in the liver of T. cruzi-infected mice. (A) The gating strategy commences with the isolation of single cells (G1). A standardized gating procedure is then applied, successively selecting CD45+Live/Dead- viable leukocytes (G2). To analyze myeloid cells based on their size (FSC) and granularity (SSC), gating is performed in G3. For the examination of inflammatory monocytes, CD11b+F4/80+ cells are gated in G4. Subsequent markers (LY6C and NOS-2) are identified as depicted in the dot plot. [file Image_3.tif]

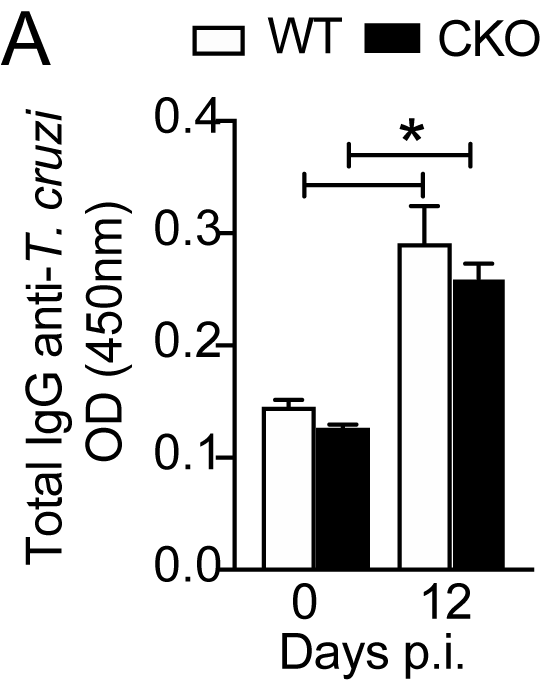

Supplement: Supplementary Figure 4 — Deletion of Blimp-1 in T cells did not change the anti-T. cruzi antibodies production during T. cruzi infection. (A) Serum anti-T.cruzi antibody level of WT and CKO mice non-infected mice and 12-day after infection with 1000 trypomastigote forms of T. cruzi Y strain. Data (mean ± SEM) are representative of two experiments with four experiments per group. Differences were analyzed with a oneway ANOVA using Tukey’s method and considered significant for *p< 0.05. [file Image_4.tif]
